# Choosing the target difference (‘effect size’) for a randomised controlled trial - DELTA^2^ guidance protocol

**DOI:** 10.1186/s13063-017-1969-5

**Published:** 2017-06-12

**Authors:** Jonathan A. Cook, Steven A. Julious, William Sones, Joanne C. Rothwell, Craig R. Ramsay, Lisa V. Hampson, Richard Emsley, Stephen J. Walters, Catherine Hewitt, Martin Bland, Dean A. Fergusson, Jesse A. Berlin, Doug Altman, Luke D. Vale

**Affiliations:** 1 Centre for Statistics in Medicine, Botnar Research Centre, Nuffield Department of Orthopaedics, Rheumatology and Musculoskeletal Sciences, University of Oxford, Nuffield Orthopaedic Centre, Windmill Road, Oxford, OX3 7LD UK; 2 Medical Statistics Group, School of Health and Related Research (ScHARR), The University of Sheffield, Regent Court, 30 Regent Street, Sheffield, S1 4DA UK; 3 Health Services Research Unit, University of Aberdeen, Health Sciences Building, Foresterhill, Aberdeen, AB25 2ZD UK; 4 Department of Mathematics and Statistics, Lancaster University, Lancaster, LA1 4YF UK; 5 Statistical Innovation Group, Advanced Analytics Centre, AstraZeneca, Riverside Building, Granta Park, Cambridge, CB21 6GH UK; 6 Centre for Biostatistics, School of Health Sciences, The University of Manchester, Jean McFarlane Building, Oxford Road, Manchester, M13 9PL UK; 7 Department of Health Sciences, University of York, Heslington, Seebohm Rowntree Building, York, YO10 5DD UK; 8 Clinical Epidemiology Program, Ottawa Hospital Research Institute, Ottawa Hospital, 501 Smyth Road, Ottawa, ON K1H 8L6 Canada; 9 Johnson & Johnson, 1125 Trenton-Harbourton Road, MS TE3-15, PO Box 200, Titusville, NJ 08560 USA; 10 Institute of Health and Society, Newcastle University, The Baddiley-Clark Building, Richardson Road, Newcastle upon Tyne, NE2 4AX UK

**Keywords:** Target difference, Clinically important difference, Sample size, Guidance, Randomised controlled trial, Pilot study, Effect size

## Abstract

**Background:**

A key step in the design of a randomised controlled trial (RCT) is the estimation of the number of participants needed. By far the most common approach is to specify a target difference and then estimate the corresponding sample size; this sample size is chosen to provide reassurance that the trial will have high statistical power to detect such a difference between the randomised groups (at the planned statistical significance level). The sample size has many implications for the conduct of the study, as well as carrying scientific and ethical aspects to its choice. Despite the critical role of the target difference for the primary outcome in the design of an RCT, the manner in which it is determined has received little attention. This article reports the protocol of the Difference ELicitation in TriAls (DELTA^2^) project, which will produce guidance on the specification and reporting of the target difference for the primary outcome in a sample size calculation for RCTs.

**Methods/design:**

The DELTA^2^ project has five components: systematic literature reviews of recent methodological developments (stage 1) and existing funder guidance (stage 2); a Delphi study (stage 3); a 2-day consensus meeting bringing together researchers, funders and patient representatives, as well as one-off engagement sessions at relevant stakeholder meetings (stage 4); and the preparation and dissemination of a guidance document (stage 5).

**Discussion:**

Specification of the target difference for the primary outcome is a key component of the design of an RCT. There is a need for better guidance for researchers and funders regarding specification and reporting of this aspect of trial design. The aim of this project is to produce consensus based guidance for researchers and funders.

**Electronic supplementary material:**

The online version of this article (doi:10.1186/s13063-017-1969-5) contains supplementary material, which is available to authorized users.

## Background

The randomised controlled trial (RCT) is widely considered to be the gold standard for assessing comparative clinical efficacy, effectiveness and safety, as well as providing an important vehicle to assess cost-effectiveness [1]. RCTs are routinely used to evaluate a wide range of interventions and have been used successfully in a variety of health care settings. Central to the design of an RCT is an a priori sample size calculation which ensures that the study has a high probability of achieving its pre-specified objectives.

A compromise is required when designing an RCT to balance the possibility of being misled by chance when there is no true difference between treatments (type I error), with the risk of failing to identify a treatment difference when one treatment is truly superior to the other (type II error) [2]. Under the conventional (sometimes referred to as *Neyman-Pearson*) approach, the probabilities of these two errors are controlled by setting the significance level (type I error) and statistical power (1 − type II error) at appropriate levels. Once these two inputs have been set, the sample size can be determined, given the magnitude of the between-group difference in the outcome that is to be detected.

The difference between groups used to calculate a trial’s sample size—that is, the ‘target difference’—is the magnitude of difference that the RCT is designed to reliably detect. It can be expressed as an absolute difference (e.g., mean difference) or a relative difference (e.g., HR or risk ratio), and it is also often referred to as the trial’s *effect size*. The required sample size is very sensitive to the target difference. Under the conventional approach, halving the target difference quadruples the sample size for a two-arm 1:1 parallel-group trial with a continuous outcome which is assumed to be normally distributed [2]. Appropriate sample size formulae vary, depending upon the proposed trial design and statistical analysis, although the overall approach is consistent. In addition to the conventional approach, other statistical approaches (to calculating the sample size) can be used, such as Fisherian/precision-based approaches, Bayesian and Bayesian decision-theoretic approaches, along with a hybrid of the Bayesian and Neyman-Pearson approaches [3–7]. However, a relatively recent review of 215 RCTs in leading medical journals identified only the Neyman-Pearson approach in use [4].

A comprehensive methodological review conducted by the original Difference ELicitation in TriAls (DELTA) group [8, 9] highlighted the available methods and limitations in current practice. It showed that despite there being many different approaches available, some are rarely used in practice [10]. Although relevant to all types of outcomes, a substantial amount of research has been carried out on patient-reported quality-of-life outcomes, reflecting not only that patients may find specifying an important difference more difficult than clinicians but also the general challenge of interpreting quality-of-life measures and the value of the patient’s perspective [11, 12]. In practice, the target difference is often not formally based upon these concepts and in many cases appears, at least on the basis of trial reports, to be determined on the basis of convenience or some other informal basis [13].

Recent surveys of practice of researchers involved in clinical trials have demonstrated that determination of the sample size, including specification of the target difference, is a more complex process than the trial reports suggest [10]. Initial guidance has been prepared for non-adaptive superiority two-arm parallel-group trials which are to be analysed according to the Neyman-Pearson approach [14]. However, this guidance does not cover trials of alternative hypotheses (i.e., equivalence/non-inferiority trials), more complex designs (e.g., multi-arm trials) or other alternative statistical approaches (Bayesian and precision-based) to choosing the target difference and reporting the sample size calculation. There are signs that the recent work led by the DELTA group has begun to influence practice through citations, presentations and anecdotal experience [15, 16]. However, it is clear that limitations in the scope and conception (because it was developed primarily for researchers) of the initial DELTA guidance mean that it does not fully meet the needs of funders and researchers in terms of understanding the role of the target difference in various designs and options available to inform its choice.

## Aim and objectives

The overall aim of the project is to produce updated guidance for researchers and funders on specifying and reporting the target difference (‘effect size’) in the sample size calculation of an RCT. The following are the specific objectives:

1. To review existing guidance provided by funders to researchers and scientific review panel/board members
2. To identify key methodological developments or changes in practice which have emerged since the comprehensive DELTA review [8, 9] was undertaken and update the DELTA method guidance
3. To determine the scope of guidance that would aid researchers and address funders’ needs
4. To achieve consensus on what structured guidance for choosing the target difference (effect size) should comprise
5. To identify future research needs

To achieve these objectives, we will systematically review the methodological literature for approaches to determining the target difference in RCTs which have been published since the DELTA review was completed in 2011 (stage 1). In addition, experts will be asked about recent methodological developments and changes in practice (stage 2). Following this, a Delphi study involving key stakeholders will be undertaken to gather views on the needed scope and focus of the guidance needed (stage 3). Embedded within the Delphi study will be a 2-day consensus workshop, which will bring together key stakeholders (stage 4) to reach agreement on key aspects of the structured guidance for researchers and funders that will be prepared. Following completion of the Delphi study, this guidance will be reviewed, finalised and disseminated (stage 5).

## Methods/design

### Overview

As noted above, we will follow a five-stage process to meet the stated project aims and objectives:

- Stages 1 and 2: conduct literature reviews and update method guidance
- Stage 3: conduct Delphi process
- Stage 4: hold a 2-day workshop and one-off stakeholder engagement sessions
- Stage 5: finalise core guidance, tailor to funding streams and disseminate to stakeholders (researchers and funders)

### Stages 1 and 2: review of methodological developments

#### Summary

A review of methodological developments will be undertaken based primarily upon an electronic search of leading journals.

#### Identifying relevant literature

The primary method for identifying reports of relevant primary and secondary research will be an electronic search in PubMed of the titles and abstracts of papers in leading journals in trials, health economics, methodology and statistics (*see* Appendix 1 for full list of journals). The set of chosen journals includes those where previous methodological work in this area has been published [8, 9], supplemented by other leading journals. Informed by the DELTA review, we will search for titles and abstracts containing the key terms ‘sample size’, ‘target difference’ and ‘effect size’, as well as common methods terms (‘important difference’). On the basis of a scoping search, the number of titles and articles identified by this search strategy varied from 9 to 45 per year, of which 3% to 15% were selected for full-text assessment. The search period will be from January 2011 (post-search period of the DELTA review) to a date 3 months prior to the consensus workshop (stage 5).

We will also review online guidance that has been provided by the relevant UK trial funding schemes run by the National Institute for Health Research (NIHR), including EME, Health Technology Assessment (HTA), the Research for Patient Benefit Programme, Programme Grants for Applied Research (PGfAR), Public Health Research (PHR), Invention for Innovation (i4i), and Health Services and Delivery Research; the Medical Research Council (MRC) Developmental Pathway Funding Scheme (DPFS); the Wellcome Trust (Health Challenge Innovation Fund); and Cancer Research UK (CRUK) (phase III clinical trial, new agent, population research). We will also review any guidance documents relating to sample size specification provided by the NIHR Research Design Service (RDS). Online guidance documents will be reviewed with individual schemed contact to provide clarification where necessary. We will also review guidance provided by leading international funding streams (National Institutes of Health [NIH], Patient-Centered Outcomes Research Institute [PCORI], Canadian Institutes of Health Research [CIHR], National Health and Medical Research Council [NHMRC]).

We shall augment the electronic journal search as follows:

1. *Contacting experts known to have an interest in the field:* We shall contact experts whom we know have an interest in the methodology of sample size calculations and specifically specifying the target difference. A number of key figures in the literature are collaborators on this project. In addition, we shall also contact authors of key studies already known to us.
2. *Methods adopted by UK clinical researchers:* As described more fully below, the Delphi process involving leading stakeholders including (UK Clinical Research Collaboration [UKCRC]) registered clinical trials units (CTUs) and MRC Hubs for Trials Methodology Research (HTMR) will provide another avenue to identifying any new methods or methodological development in methods previously identified.

#### Screening and assessing papers for inclusion and summarising findings

Papers reporting a methodological development for specifying the target difference for a trial will be included. Titles and abstracts will be screened independently by two people. The full-text papers will be obtained if on initial screening they are considered potentially relevant. Only those papers deemed relevant after this will be included in the review.

#### Selection of methods

Methodological developments will be assessed by two reviewers and noted according to the categorisation used in the previous review. A third (content expert) member of the team will act as arbiter if there is disagreement at any stage.

#### Reporting

Each innovation will be summarised in turn and placed in the context of the existing guidance. An updated narrative summary of the evidence for each method will be produced accordingly as appropriate.

### Stage 3: Delphi study

#### Summary

We will conduct a multi-round (at least two and no more than three rounds) Delphi study with stakeholders known to have an interest in the design of RCTs about guidance for specifying the target difference in an RCT sample size calculation. The Delphi study will have embedded in it a 2-day consensus meeting and one-off stakeholder engagement sessions (stage 4; *see* below for details). Findings from the first Delphi round will feed into the 2-day consensus meeting, which in turn will inform the subsequent questionnaires.

#### Participants

Invitations will be sent to known experts (informed by the DELTA review and stage 1) along with representatives of key trial groups. One named individual per group (unit, board, MRC HTMR, RDS centre, or programme; e.g., the director, chair or senior methodologist) will be invited to participate. Groups which will be invited to send representatives to participate will include the UKCRC network of clinical trial units (CTUs), the MRC HTMRs, NIHR/MRC/CRUK funding programme panels, the NIHR statistics group and the NIHR RDS. They will be contacted using publicly available contact information. These groups represent UK centres and networks of excellence that undertake high-quality trials research. As of 1 July 2016, there are 48 (fully or provisionally) registered units, five MRC HTMRs and the ten regions in the NIHR RDS in England and the Research Design and Conduct Service in Wales. (Analogous services do not exist for Scotland and Northern Ireland). 

To give an additional perspective, we will also the organising committee of the NIHR statistics group, to participate as stakeholders in the Delphi process.

#### Sample size

It is anticipated that around one-third of invitees will agree to participate in the Delphi process. To achieve a minimum of 30 participants, at least 90 invitations will need to be sent out, though no strict maximum will be applied to reflect the arbitrary nature of this target.

#### Methods

An initial invitation email will be sent to potential participants. If they agree to participate, they will be entered into the Bristol Online Surveys (BOS) system online, which will then administer the separate questionnaire rounds. A separate email will be sent to each participant with a personalised link enabling access to the online questionnaire and allowing completion. The DELTA^2^ survey rounds will be administered online using the BOS system (University of Bristol). Participants will be invited by email to participate in an online questionnaire and assess the importance of potential areas to cover topic items selected from previous research.

#### Content of the questionnaires

The initial round 1 questionnaire will ask for information relating to the background of the individual in terms of training, role and experience. Questions will be tailored to the stakeholder groups with some questions addressed only to specific stakeholders (e.g., more methodologically focussed questions for researchers in the area). The questionnaire will also ask about the type of trials (e.g., in terms of phases), sample size approaches (e.g., Bayesian, Bayesian decision-theoretic), designs (e.g., cluster, adaptive) and associated considerations aspects (e.g., missing data and compliance) that should be covered by future guidance. The survey, together with stakeholder meetings, will identify the key topic areas and also views on scope. An opportunity to raise an additional topic or to make a general comment on guidance in this area will be provided.

The round 1 questionnaire (Additional file 1: Appendix 4) is anticipated to take approximately 10–15 minutes to complete. Subsequent rounds (the second and, if necessary, third questionnaires) will be of a similar nature (some questions will be the same, whereas others will be related questions of a similar style and topic) and length (again taking approximately 10–15 minutes to complete), and they will include a summary of findings from the previous rounds. As necessary, we will use a structured telephone discussion to elicit further details (if permission is granted within the questionnaire).

#### Data collection and analysis

Responses are stored securely on the BOS system and will be downloaded to a secure file space. Analyses of findings will be summarised both overall and by stakeholder group. Where appropriate, an ordinal 5- or 6-point scale (e.g., ‘Strongly disagree’ to ‘Strongly agree’) will be used, which includes ‘neutral’ and ‘no opinion’ options where appropriate. Similarly, a scale ranging from ‘none’ to ‘extensive’ will be used to assess the degree to which an issue or type of design needs to be covered in any future guidance. All analyses will be descriptive in nature, and no inferential statistical analyses are planned (i.e., no statistical hypotheses will be formally tested).

#### Output

It is anticipated that the findings of the Delphi study will be summarised and submitted for publication as a peer-reviewed manuscript.

### Stage 4: 2-day consensus meeting and one-off stakeholder engagement sessions

#### Summary

In addition to the Delphi process, we will involve stakeholders through one-off events as part of the consensus-building process. The main way this will occur is through a face-to-face 2-day consensus meeting of approximately 30 stakeholders to agree on the structure and content of the guidance to be provided with post-meeting review and refinement (stage 5). Additionally, we will hold one-off engagement sessions at relevant stakeholder meetings. Further details pertaining to participants and content of the 2-day consensus meeting and the one-off engagement sessions are given below.

#### 2-day consensus meeting

Meeting participants will be selected to cover a range of perspectives, expertise levels and roles. Draft guidance and recommendations for researchers and funders of clinical trials will be developed, incorporating previous work updated in light of the initial findings from stage 3.

The structure of the 2-day meeting will be informed by stages 1–3 of the DELTA^2^ project and discussion with stakeholders. The workshop will likely include presentations of the previous DELTA project and how this has been updated in light of stages 1 and 2 of the DELTA^2^ project, along with findings from the first round of the stage 3 Delphi study. Parallel small-group sessions will be considered to increase available time and enable more technical topics (e.g., statistical approach and design-specific issues) to be covered. The guidance will concisely detail the strengths and weaknesses of each approach and will be divided into separate guidance sections on methods, study design-specific issues (e.g., adaptive trials) and special topics (e.g., types of outcome and summary measure).

#### One-off stakeholder engagement sessions

One-off stakeholder engagement sessions will include contributing session proposals to relevant conferences such as the SCT and PSI, as well as holding a meeting with the Medical Section of the Royal Statistical Society, to enable a broader group of stakeholders to contribute to the consensus-building process. Participants in the one-off sessions will reflect the membership of the relevant group and will be somewhat opportunistic. Content will reflect the current stage of consensus-building and, where relevant, findings from the Delphi process and draft guidance.

### Stage 5

#### Tailoring the guidance

Following the completion of stages 1–4, provisional guidance will be drafted and circulated to the project team and consensus meeting participants for comment (stage 5). Once the core guidance is agreed upon, we will approach the trial-relevant MRC/NIHR funding panels as per stage 2 to ensure that the guidance meets each funding programme’s needs. We will engage with the individual UK funding bodies to tailor guidance to a format that they would find most useful.

#### Identifying future research needs

As part of the development of the guidance and recommendations, key uncertainties that remain will be recorded, thus enabling further research to address them to be prioritised.

## Discussion

Researchers face a number of difficult decisions when designing an RCT, including the choice of trial design, primary outcome and sample size. The latter is driven largely by the choice of target difference (‘effect size’), although other aspects of sample size determination also contribute. Existing guidance on determination of the target difference is limited, and there has been growing recognition of the need for greater guidance for funders and researchers, as well as other key stakeholders, such as patients and the respective clinical communities. DELTA^2^ is seeking to produce practical and comprehensive guidance which is applicable to the vast majority of trials to bridge the gap between existing guidance and the needs of researchers.

## Appendix 1: list of journals to be reviewed, by subject area

Trials (*Trials*, *Clinical Trials*, *Contemporary Clinical Trials*)

Health economics (*Journal of Health Economics*, *Health Economics*, *Value in Health*, *European Journal of Health Economics*, *International Journal of Epidemiology*, *Medical Decision Making*, *Pharmacoeconomics*, *Public Health*),

Methodology (*American Journal of Epidemiology*, *American Journal of Public Health*, *BMC Medical Research Methodology*, *Epidemiology*, *Journal of Clinical Epidemiology*, *International Journal of Epidemiology*)

Statistical analysis (*Biometrics*, *Biometrika*, *Biostatistics*, *Biometrical Journal*, *Journal of the Royal Statistical Society. Series C: Applied Statistics*, *Statistics in Biopharmaceutical Research*, *Statistics in Medicine*, *Statistical Methods in Medical Research*, *Journal of Biopharmaceutical Statistics*, *Pharmaceutical Statistics*)

## Appendix 2: list of funding body guidance to be reviewed

***United Kingdom:*** NIHR (Efficacy and Mechanism Evaluation [EME], Health Technology Assessment [HTA], Research for Patient Benefit Programme [RfPB], Programme Grants for Applied Research [PGfAR], Public Health Research [PHR], Invention for Innovation [i4i], Health Services and Delivery Research); MRC (Developmental Pathway Funding Scheme [DPFS], Wellcome Trust [Health Challenge Innovation Fund], Arthritis Research UK, British Heart Foundation, Cancer Research UK [clinical research, new agent, population research]); NIHR Research Design Service; NIHR Statistics Group; and NHS Health Research Authority (HRA).

***United States:*** Food and Drug Administration, PCORI, National Institutes of Health, and Agency for Healthcare Research & Quality.

***Canada:*** Health Canada (drugs and health products) and Canadian Institutes of Health Research (CIHR).

***Other:*** European Commission (Horizon 2020) and Australian Clinical Trials.

## Appendix 3: sample search strategy

Sample PubMed search: (‘sample size’[TIAB] OR ‘target difference’[TIAB] OR ‘effect size’[TIAB] OR ‘important difference’[TIAB] or ‘detectable difference’[TIAB] OR ‘power calculation’[TIAB] OR ‘value of information’[TIAB] OR ‘value of perfect information’[TIAB] OR ‘value of partial perfect information’[TIAB] OR ‘value of sampling information’[TIAB] OR ‘expected net gain’[TIAB]) AND ‘Trials’[TA] AND (‘2011/01/01’[PDAT]: ‘2016/03/31’[PDAT]).

## Additional file

Additional file 1: Appendix 4 Delphi online questionnaire images. See accompanying document with the filename DELTA2_Protocol_Appendix4_DelphiQuestionnaire_11Jul2016.pdf (PDF 108 kb)

## Abbreviations

BOS: Bristol Online Surveys
CIHR: Canadian Institutes of Health Research
CRUK: Cancer Research UK
CTU: Clinical Trials Tnit
DELTA: Difference ELicitation in TriAls
DPFS: Developmental Pathway Funding Scheme
EME: Efficacy and Mechanism Evaluation
HTA: Health Technology Assessment
HTMR: Hubs for Trials Methodology Research
i4i: Invention for Innovation
MRC: Medical Research Council
NHMRC: National Health and Medical Research Council
NIH: National Institutes of Health
NIHR: National Institute for Health Research
PCORI: Patient-Centered Outcomes Research Institute
PGfAR: Programme Grants for Applied Research
PHR: Public Health Research
PSI: Statisticians in the Pharmaceutical Industry
RCT: Randomised controlled trial
RDS: Research Design Service
SCT: Society for Clinical Trials
UKCRC: UK Clinical Research Collaboration
