# Supplementary material for: Choosing the target difference (‘effect size’) for a randomised controlled trial - DELTA2 guidance protocol
Source: Trials. 2017 Jun 12;18:271. doi: 10.1186/s13063-017-1969-5 (PMC5469157; doi:10.1186/s13063-017-1969-5)
Supplement: Additional file 1: — Appendix 4 Delphi online questionnaire images. See accompanying document with the filename DELTA2_Protocol_Appendix4_DelphiQuestionnaire_11Jul2016.pdf (PDF 108 kb) [file 13063_2017_1969_MOESM1_ESM.pdf]

DELTA2 Survey

Page 1: Introduction

Randomised controlled trials (RCT) are widely considered to be the optimal study design to assess comparative clinical efficacy and effectiveness, along with the cost implications of health interventions. Central to the validity of a RCT, is an *a-priori* sample size calculation which ensures the study has a reasonable chance to achieve its pre-specified objectives. Typically the sample size is calculated in order to ensure it is likely that a particular the magnitude of a difference between groups ("target difference" or "effect size") will be detectable.

Current published guidance on specifying the target difference is limited. See for example (Cook et al.) which covers only standard (superiority two-arm parallel) group trials and does not address Bayesian approaches or more complex trial designs (e.g. multi-arm trials).

The role of this survey is to determine the scope of guidance that researcher's and funders would find useful.

By completing this short survey (10 questions), which takes around 10 min to complete, you will be helping shaping the guidance.

Thank you for your time and support, it's greatly appreciated!

I confirm I am over 18 years old and agree to take part in this study.

- ○ Yes
- ○ No

Page 2: About you

Your role in RCTs (select all that apply): ✱ *Required*

Please select at least 1 answer(s).

- ☐ Involved in RCT design (Lead/Chief Investigator)
- ☐ Involved in RCT design (Statistician/Methodologist)
- ☐ Involved in RCT design (Collaborating Clinician)
- ☐ Involved in analysis of RCTs
- ☐ Serves on a funding panel/board which evaluates applications for RCT funding
- ☐ Other (Please specify)

If you selected Other, please specify:

____

Primary RCT related affiliation: ✱ *Required*

- ○ Academic institution
- ○ Healthcare provider (e.g. NHS in the UK)
- ○ Funder of RCTs (e.g. NIHR in the UK or NIH in the US)
- ○ Pharmaceutical/medical device company
- ○ Contract research organsiation
- ○ Patient and public representative
- ○ Other (Please specify)

If you selected Other, please specify:

____

Where do you work? If you work across Europe or Internationally please choose the category in which the majority of your work is performed. *

Required

- UK
- Ireland
- Other European Country
- US
- Canada
- Australasia
- Other (Please specify)

If you selected Other, please specify:

Page 3: Scope of guidance

Types of studies

Guidance for specifying the target difference for a phase III/IV (often called “definitive” or “confirmatory”) trial needs to be dealt with separately from early phase, pilot or feasibility trials.

	Degree of opinion ✱ *Required*					
	No opinion	Disagree strongly	Somewhat disagree	Neutral	Somewhat agree	Agree strongly
Choose one	☐	☐	☐	☐	☐	☐

Methods for specifying target difference

Should the following approaches be considered a formal method and covered within the guidance?

More info

	Degree of opinion ✱ *Required*					
	No opinion	Disagree strongly	Somewhat disagree	Neutral	Somewhat agree	Agree strongly
“Standardised effect size” (further information available above)	☐	☐	☐	☐	☐	☐
“Value of information” (further information available above)	☐	☐	☐	☐	☐	☐

Page 4: Scope of guidance

Special topics

Degree of coverage required for the following special topics in relation to specification of the target difference:

	Level of guidance required ✱ *Required*				
	No Opinion	None	Brief	Proportionate	Extensive
Alternative research questions (e.g. equivalence and non-inferiority)	○	○	○	○	○
Bayesian approaches	○	○	○	○	○
Health economic outcomes/Cost effectiveness	○	○	○	○	○
Mechanistic (compliance analyses) studies	○	○	○	○	○
Missing data	○	○	○	○	○
Multiple primary outcomes	○	○	○	○	○
Target difference choice in relation to interim analyses	○	○	○	○	○
Public and patient perspectives	○	○	○	○	○
Other (please detail below)	○	○	○	○	○

Other special topics with brief reason for inclusion.

Page 5: Scope of guidance

Complex designs

Degree of coverage for trials with more complex designs:

	Level of guidance required ✱ *Required*				
	No Opinion	None	Brief	Proportionate	Extensive
Adaptive designs	○	○	○	○	○
Cluster randomised trials	○	○	○	○	○
Cross-over designs	○	○	○	○	○
Factorial designs	○	○	○	○	○
Multi-arm (excluding factorial) designs	○	○	○	○	○
Multi-stage (including dynamic treatment regime) designs	○	○	○	○	○
Stepped wedge designs	○	○	○	○	○
Within subject paired (e.g. eyes/split mouth) designs	○	○	○	○	○
Other (please specify below)	○	○	○	○	○

Other designs with reason for inclusion.

Page 6: Existing guidance

Please briefly review the existing guidance paper on this topic.

Specifying the target difference in the primary outcome for a randomised controlled trial: guidance for researchers (Cook et al. 2015).

The existing paper is useful: ✱ *Required*

Please don't select more than 1 answer(s) per row.

Please select at least 1 answer(s).

	Disagree strongly	Somewhat disagree	Neutral	Somewhat agree	Agree strongly
Choose one:	☐	☐	☐	☐	☐

How could it be improved?

Page 7: Additional comments

Are there any other comments related to guidance on specifying target difference for randomised trials which you would like to make?

Page 8: Thanks

Thanks for completing this survey which is very helpful and greatly appreciated!
